# Effects of brief bouts of exercise, embodied cognitive training, and their combination on social anxiety in rural left-behind children: a randomized controlled trial

**DOI:** 10.3389/fpsyg.2026.1733845

**Published:** 2026-03-17

**Authors:** YiPing Luo, JiaXi Chen, Qian Yang, XiaoLin Li, Xiao Liu, WeiXin Dong, Jun Chen, ChunXia Lu

**Affiliations:** Department of Sport Education, Hunan Normal University, Changsha, Hunan, China

**Keywords:** brief bouts of exercise intervention, combined intervention, embodied cognitive training intervention, rural left-behind children, social anxiety

## Abstract

**Objectives:**

This study aimed to examine the effects of three intervention modes on social anxiety among rural left-behind children in China, including brief bouts of exercise, defined as short, structured periods of physical activity integrated into daily routines; embodied cognitive training, defined as training that integrates cognitive tasks with goal-directed bodily movements; and a combined intervention incorporating both approaches, with outcomes assessed at baseline, post-intervention, and follow-up.

**Methods:**

A randomized controlled trial (RCT) was conducted to examine the effects of different interventions on social anxiety among rural left-behind children. Participants were recruited purposively from one rural primary school in Shaodong City, Hunan Province, and then randomly allocated to four groups (*n* = 25 each): brief bouts of exercise (BBEG), embodied cognitive training (ECG), combined intervention (CIG), or control (CG). Demographic information was collected using a researcher-designed questionnaire. The interventions were delivered over a 12-week period, with all intervention groups receiving their respective interventions four times per week, followed by a 6-week follow-up. Social anxiety was assessed using the self-report Social Anxiety Scale for Children at baseline, immediately post-intervention, and follow-up. Outcomes were analyzed using repeated-measures ANOVA to examine group, time, and interaction effects.

**Results:**

At baseline, no significant differences were observed among groups in social anxiety (SA), fear of negative evaluation (FNE), or social avoidance and distress (SAD) (all *p* > 0.05), indicating good baseline comparability. Mixed-design analyzes of variance revealed significant Group × Time interactions across all outcome measures (*p* < 0.05), indicating that changes over time differed significantly across groups. From pre-test to post-test, participants in the brief bouts of exercise group, embodied cognitive training group, and combined intervention group showed significant reductions in SA, FNE, and SAD (*p* < 0.001), whereas no significant changes were observed in the control group (*p* > 0.05). These improvements were maintained at follow-up (*p* < 0.01). *Post hoc* comparisons indicated that, at both post-test and follow-up, all intervention groups demonstrated significantly greater symptom reductions than the CG (*p* < 0.05). Notably, the CIG exhibited significantly greater improvements than both single-intervention groups across all outcomes, and the BBEG achieved greater reductions than the ECG (*p* < 0.05).

**Conclusion:**

All three interventions were associated with reductions in social anxiety relative to the control group. Compared with single-modality interventions, the combined intervention demonstrated greater improvements during the intervention period and follow-up.

## Introduction

With the rapid urbanization of developing countries, the number of migrant workers has increased substantially. However, due to institutional and economic constraints, many migrant workers cannot bring their children to the cities where they work, leading to a large population of left-behind children (LBC) in rural China (Liu et al., 2020). Ample evidence suggests that LBC experience poorer mental health than their non-left-behind peers (Wen and Lin, 2012). Showing higher levels of depression and anxiety (Dai and Chu, 2018), and greater vulnerability to suicidal behaviors (Xiao et al., 2019). These psychological difficulties not only hinder their healthy development but also pose potential risks to social stability.

Social anxiety (SA), defined as an excessive fear of negative evaluation in social situations (Rapee and Heimberg, 1997) is characterized by tension, fear, and avoidance of social interactions (Holas et al., 2023). Evidence indicates that SA is one of the most common psychological problems among LBC in China. If left unaddressed, childhood SA may increase the risk of later depression, substance use, and impaired occupational functioning (Zeytinoglu et al., 2022). These findings highlight the urgent need for effective, scalable interventions that can be feasibly implemented in this vulnerable group.

Existing interventions for anxiety management include psychological and pharmacological treatments. Cognitive and relaxation-based therapies are well established, and meta-analyzes have confirmed their effectiveness in reducing stress, anxiety, and depression (Khir and Yunus, 2023; Markowitz et al., 2014). However, some individuals show limited or no response to cognitive-behavioral therapy (CBT), particularly those with chronic anxiety or specific personality traits, suggesting that CBT alone may not be sufficient for all cases (Hofmann et al., 2012; Loerinc et al., 2015). Behavioral approaches such as attentional training and physical exercise have also been applied with varying success (Biddle and Asare, 2011), as attention to bodily sensations and physical activation can help regulate sympathetic arousal and alleviate anxiety (Papola et al., 2024; Weng et al., 2021). Recent child-focused research has emphasized that physical activity in middle childhood operates through multidimensional developmental pathways, including physical competence, motivation, and confidence, often conceptualized within physical literacy frameworks (Hadier et al., 2024, 2025). Yet, single-modality interventions such as mindfulness-based stress reduction (Grossman et al., 2004; Strauss et al., 2014) or dance therapy (Koch et al., 2014) often yield inconsistent outcomes, underscoring the need for integrative approaches.

In recent years, embodied cognition training (ECT) has emerged as a promising intervention that combines bodily movement with cognitive engagement. Rooted in embodied cognition theory, which posits that human cognition arises from the dynamic interaction between the brain, body, and environment (Davis and Markman, 2012; Gallagher, 2006). ECT has been shown to enhance bodily awareness, emotion regulation, and social attunement, leading to reductions in social anxiety (Loew et al., 2006). At the same time, the concept of “brief bouts of” (brief, intermittent bouts of moderate-to-vigorous activity) has gained attention for its time efficiency and accessibility. Evidence suggests that these short physical activities can reduce anxiety through catecholamine release (Lin and Gao, 2023), improve socanxiety (Zhange) in youth (Landry and Driscoll, 2012), and decrease test anxiety (Zhang et al., 2022). Notably, brief bouts of exercise overcome common barriers such as time, cost, and facility limitations, making them easily adaptable to school and home environments (Weston et al., 2025).

Accordingly, this study aimed to compare the effects of brief bouts of exercise, embodied cognitive training, and a combined intervention on social anxiety among rural left-behind children. Using a 12-week intervention with a 6-week follow-up, changes in total social anxiety, fear of negative evaluation, and social avoidance and distress were examined across baseline, post-intervention, and follow-up assessments. It was hypothesized that all intervention groups would show greater reductions in social anxiety than the control group, and that the combined intervention would produce larger and more sustained improvements than either single-modality intervention.

## Methods

### Study design, setting, and sampling approach

This study adopted a randomized controlled trial (RCT) design to examine the effects of brief bouts of exercise, embodied cognitive training, and their combined intervention on social anxiety among rural left-behind children. The study was conducted at Huangdu Primary School in Shaodong City, Hunan Province, China. The interventions were delivered over a 12-week period, with all intervention groups receiving their respective interventions four times per week, followed by a 6-week follow-up.

Participants were recruited using purposive sampling from eligible students in Grades 4 and 5 (10.36 ± 0.86 years). After eligibility screening and completion of baseline assessments, participants were randomly allocated to one of four groups using a computer-generated block randomization scheme (1:1:1:1).

### Sample size and participants

**Figure 1:**
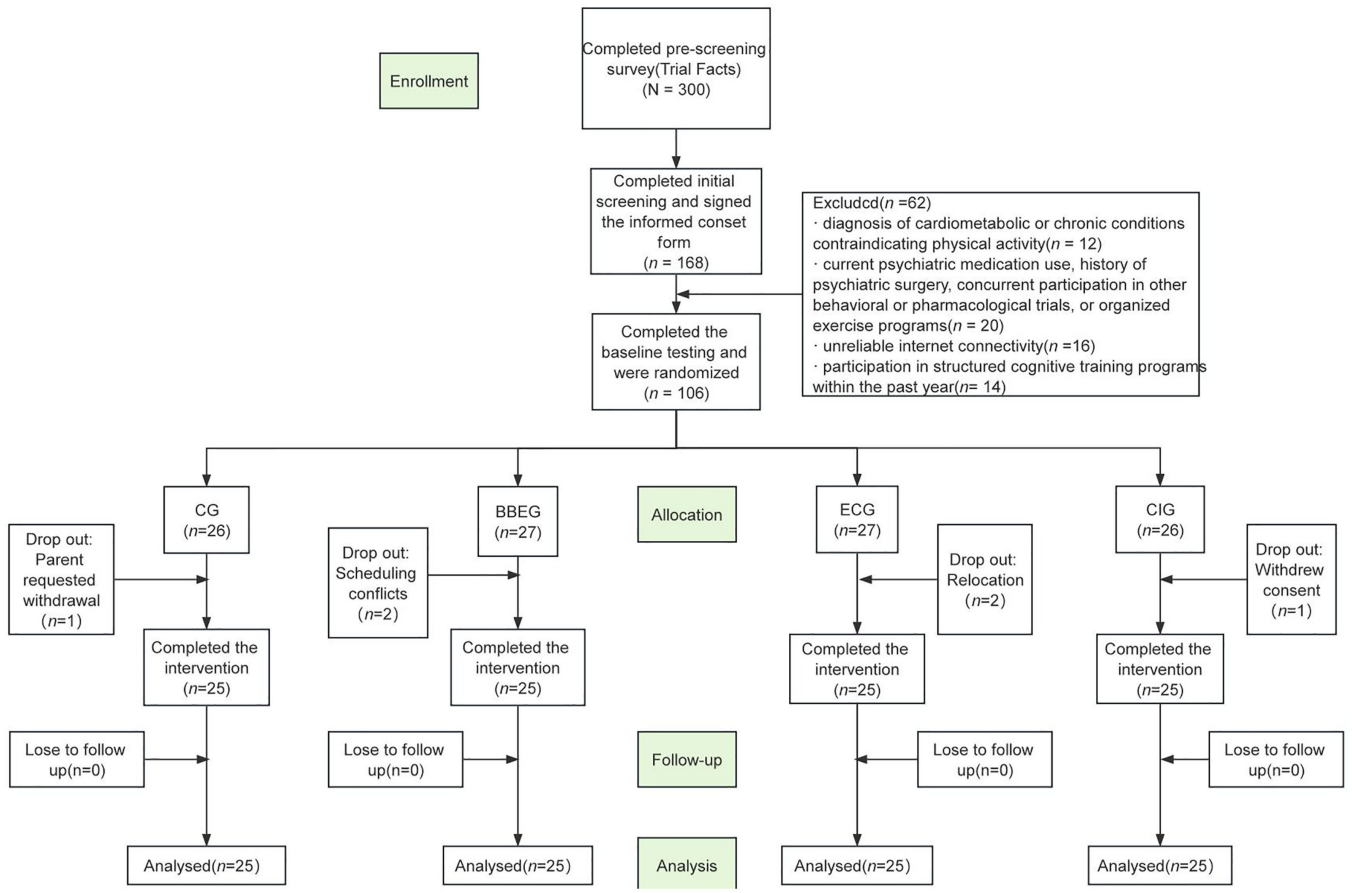
Flow diagram of participant recruitment and allocation.

*A priori* power analysis was performed using G*Power 3.1 to determine the minimum sample size required for a repeated-measures analysis of variance (ANOVA), which was designed with four groups and three time points. Effect size benchmarks were adopted in line with Cohen (2013), who defined small (*f* = 0.1), medium (*f* = 0.25), and large (*f* = 0.4) effect sizes. Given the complexity of human behavior and potential confounding variables, small-to-medium effect sizes are commonly reported in relevant studies (Faul et al., 2007); thus, a small-to-medium effect size (*f* = 0.3) was assumed for the analysis. Other key parameters were set as follows: statistical power was set to 80%, and the significance level (*α*) was set to 0.05. The power analysis results indicated that a minimum of 88 participants were required to meet these criteria. To account for potential participant attrition, a 20% attrition rate was anticipated, consistent with the attrition rates reported in previous behavioral and cognitive intervention studies (Cavanagh et al., 2013; Khoury et al., 2013; Raes et al., 2014). This adjustment increased the target sample size to 106. However, six participants withdrew prior to the completion of the intervention, with reasons including relocation, withdrawal of consent, scheduling conflicts, and withdrawal requested by parents. Consequently, the final analytic sample comprised 100 participants, with an equal distribution of 25 participants per group. The flow of participants throughout the trial is illustrated in Figure 1.

### Eligibility criteria

Eligibility criteria: inclusion criteria were as follows: (i) enrollment in Grade 4 or 5; (ii) a score ≥ 8 on the Social Anxiety Scale for Children (La Greca and Lopez, 1998); (iii) regular access to a computer, tablet, or smartphone; and (iv) classification as left-behind children (defined as residing in rural households while one or both parents have worked away from home for at least six consecutive months).

Exclusion criteria included: (i) diagnosis of cardiometabolic or chronic conditions contraindicating physical activity (e.g., heart disease, diabetes); (ii) current psychiatric medication use, history of psychiatric surgery, concurrent participation in other behavioral or pharmacological trials, or organized exercise programs; (iii) unreliable internet connectivity; and (iv) participation in structured cognitive training programs within the past year.

### Study procedure

This randomized controlled trial evaluated the feasibility and efficacy of three intervention conditions: brief bouts of exercise, embodied cognitive training, and a combined intervention. The study comprised five sequential phases: (i) screening and enrollment, (ii) baseline assessment, (iii) intervention implementation with coordinated home–school support, (iv) monitoring of intervention adherence and intensity using wearable devices, and (v) post-intervention and follow-up assessments. Teachers supervised school-based sessions, while parents supported home-based activities and recorded adherence. The research team oversaw intervention delivery, participant communication, and data management. Allocation concealment was ensured using sequentially numbered, sealed, opaque envelopes prepared by an independent study coordinator. During school hours, teachers supervised the intervention sessions, while parents facilitated home-based activities and maintained daily logs of adherence. The research team consisted of a senior professor with expertise in exercise rehabilitation and clinical psychology, assisted by three graduate students majoring in exercise science and three in psychology, who were responsible for intervention monitoring, participant communication, and data management. On a different day, participants were introduced and familiarized with the study-specific online platform and were guided through the account login and initialization process. Data were collected online using a secure survey platform. Participants accessed individualized survey links distributed via email or instant messaging, which were restricted to single use to prevent duplicate entries. To enhance data quality, items were mandatory, time-stamped automatically, and accompanied by standardized instructions. Research staff provided real-time support through an online help channel to address technical issues.

### Intervention

#### Brief bouts of exercise group (BBEG)

The BBEG received the intervention remotely via a mobile AI-assisted exercise platform (Tiantian Tiaosheng, V4.0.89–7,812, China). The platform provided standardized instructional videos and integrated automated movement recognition, repetition counting, and real-time feedback for selected activities. Gamified features and data-tracking functions were incorporated to enhance engagement and support adherence throughout the intervention period. Based on the platform’s built-in exercise resources, the intervention was structured into five training modules: stretching, lower-body, full-body, core, and upper-body training. During each training session, participants were required to complete all five modules, with four exercises selected ad libitum from each module to ensure comprehensive yet flexible training. The intervention lasted 12 weeks, with a prescribed frequency of four sessions per week. In each session, physical activity was performed as multiple brief bouts that were continuously accumulated within the same day to achieve a total exercise duration of at least 40 min, rather than as a single continuous bout. Individual bouts typically lasted 5 to 10 min, with short, self-paced inter-bout intervals permitted.

The program followed a three-phase progressive structure, with exercise intensity guided by relative heart rate and perceived exertion. Phase 1 (Weeks 1–4) targeted 65–70% of predicted HR_max and ratings of perceived exertion (RPE) of 12–13, using low-complexity rhythmic movements such as small step activation, high knees, jumping jacks, and basic jump rope. Phase 2 (Weeks 5–8) targeted 70 to 75% HR_max and RPE 14–15, using moderate complexity movements such as squats, dynamic squats, alternating side lunges, and plank holds. Phase 3 (Weeks 9–12) targeted 75–80% HR_max and RPE 16–17, using higher intensity routines available in the app, such as mountain climbers, touch high jumps, star jumps, and obstacle jumps. Participants were instructed to adjust movement pace, range of motion, or exercise selection to remain within the target intensity zones. No equipment-based exercises were included.

**Figure 2:**
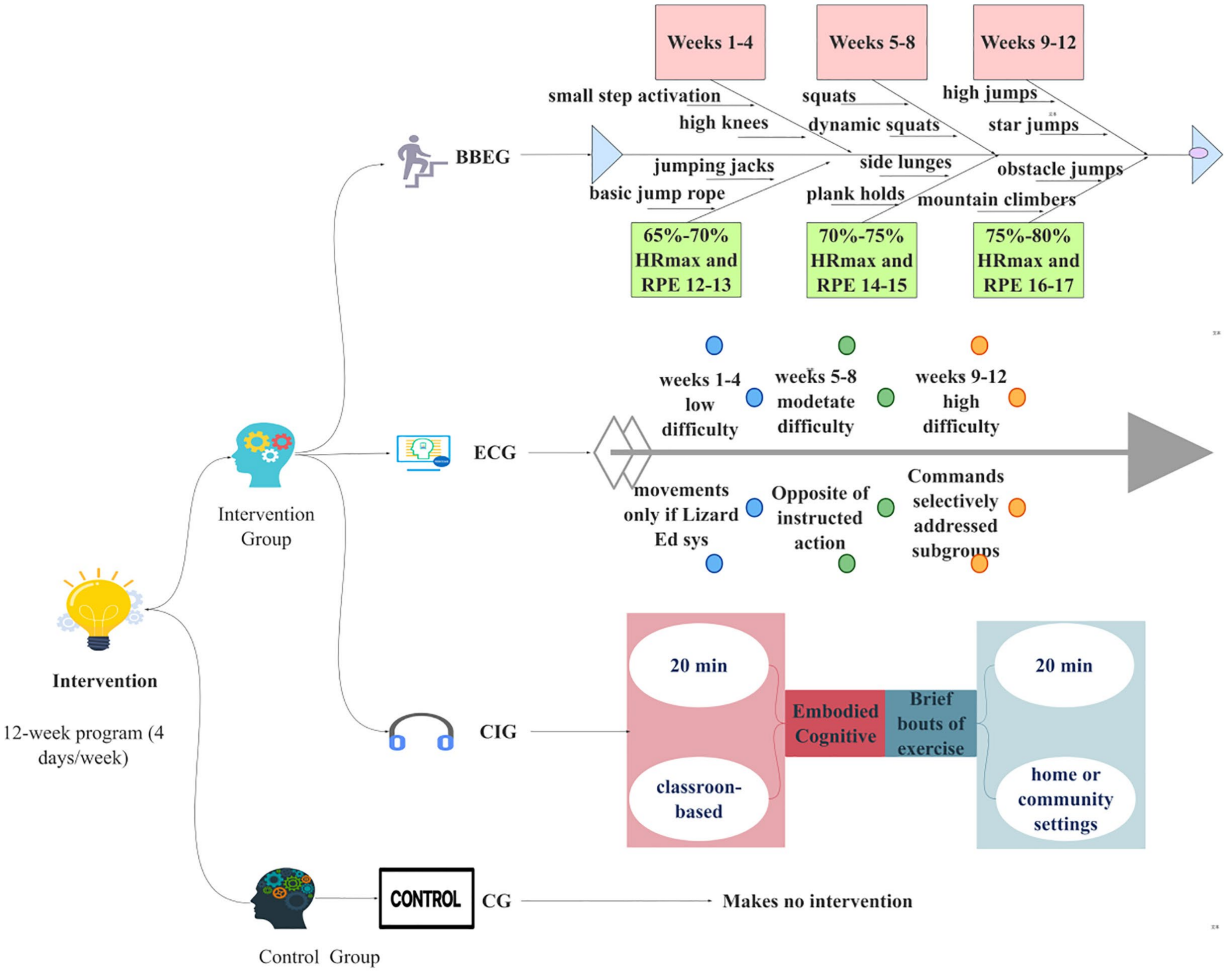
Overview of the intervention structure.

Adherence to home-based exercise was monitored using platform-generated activity logs that recorded completed exercises and accumulated session duration. These records were supplemented by parent-assisted confirmation and post-session ratings of perceived exertion using the Borg 6–20 scale (Borg, 1998). To support feasibility, wearable heart rate monitors and accelerometers were applied only in a subsample of participants during prespecified monitoring weeks to verify exercise implementation and intended intensity, whereas platform logs were used to quantify adherence across the full intervention period. Group-level physical activity validation indicators are reported in Supplementary Table S1, and the platform interface and exercise library are illustrated in Supplementary Figures S2, S3 (see Table 1; Figure 2).

**Table 1:** Description of selected actions in the five platform-based training modules.

Serial number	Module name	Content, substance	Interaction rules
Module 1	Stretching training module	Sit-and-reach	Sit sideways on the screen, straighten your legs, and bring your feet together. Place your palms facing down and extend your arms together toward your toes. The upper body is flexed forward, and the fingers move forward at a constant speed until reaching the farthest distance.
		Big Pinwheel	The whole body appears on the big screen. Keep your hands reaching for the screen while making the ‘big’ gesture. While maintaining the tension of the waist and back, touch the opposite toe with your hand.
		Sitting on the footrest	Facing the screen, sitting on the floor. Place your feet together with the soles facing each other and the balls of your feet close to your hips. Keep your back straight and place your hands lightly on your knees or your back. Press down on the knee to feel the tension between the legs.
		Little swallow flying	Move at a steady pace, not hold on for too long.
		Gentle back stretch in a kneeling position	Lie down in a kneeling position with your body naturally relaxed. Extend your arms forward and lift your hips. Press down on the shoulders to feel the stretch in the lower back.
		One-leg deadlift	Stand upright, shift your weight to the left, and lift one leg backward. Keep your shoulders, hips, knees, and ankles in a straight line parallel to the ground, with your hands hanging naturally at your sides. During the recovery phase, the body and legs return to their original positions.
Module 2	Lower limb training module	Small step activation	Step into the green circle, stand with feet slightly wider than shoulder-width apart, and squat down to lower your center of gravity. Stabilize your upper body and use your thigh and buttock muscles to quickly move your legs across the mat. The forefoot lightly touches the ground and then quickly bounces up.
		Pile driving	Move quickly sideways to the green marker that appears on the screen. Raise your knees and legs high, then gently step down onto the post. Keep a safe distance to ensure your whole body is within the screen.
		Quick kick	Face the screen, lower your center of gravity, and focus. Quickly kick the green ball that appears on the screen and avoid the red ball. Keep a safe distance to ensure your whole body is within the screen.
		*In situ* box jump	Stand in the middle of the screen where the box is. The jump and leg recovery exceed the height of the box. The leg movement is completed in a coherent and rhythmic manner.
		Deep knee bend	Stand relaxed in front of the screen, with chest out, head up, waist tight, and abdomen in. When the screen displays a small bench, move to the corresponding position to perform squats. Keep your thighs parallel to the ground when squatting. Keep your whole body within the screen, and avoid standing too close or too far away.
		Obstacle jump	Engage your entire body and jump left and right to enter the green circle. Keep the body flexible, jump and retract, and pay attention to balance. You can use your arms to lift your body off the ground.
Module 3	Full-body training module	Jumping	Stand up straight with your feet in the two squares in the middle of the screen. Jump, spread your legs, move to the squares on the left and right, and raise your hands above your head. Jump again and return to the initial action. Keep your whole body within the screen.
		Bob	Stand sideways and enter the screen with your whole body. After starting, quickly brace yourself on your hands and knees, then straighten up by pulling your legs together.
		Sequential ball picking	Stand with feet shoulder-width apart, knees slightly bent, and hands placed on your chest. Focus your attention and look at the ball that appears on the screen. Touch the target balls in numerical order from smallest to largest.
	
		Starfish jump	Enter the screen. With a slight bend of the knees, the athlete exerts force to jump and extends their limbs in the air. Raise both hands above your head. The opening range of both hands and feet should be greater than shoulder width.
		Balance stand	The entire body appears in the center of the screen. Stand on one leg and lift one leg backward. Straighten both legs to earn bonus points.
Squat with twist and punch	After squatting, quickly stand up and smash the ice.
Module 4	Core training module	Plank	The shoulder, hip, knee, and ankle joints are aligned in a straight line. Keep your upper arm vertical and your forearm flat on the floor. The toes are forcefully hooked forward, with all ten toes exerting steady force.
		Hip bridge	Lie flat on the mat, bend your knees, and enter the screen from the side. Push up your waist with your hips. The torso and thighs are aligned on the same plane.
		Sit-up, situp, abdominal curl	Lie flat on your side with knees bent, perform sit-ups, and use abdominal strength to sit up. Reply to the lying flat position before performing the next action. Do not support your body with your arms and keep your legs fixed.
		V-leg raise	Lie flat on the mat and use your abdominal muscles to maintain a V-shaped posture. Focus on exerting force through the abdominal muscles, with hands providing auxiliary support to maintain body stability. Keep your legs straight and lift them up.
		Static hip bridge	Lie flat on the mat, bend your knees, and enter the screen from the side. Push up your waist with your hips. The torso and thighs are aligned on the same plane.
		Supine leg lift	Stay on the mat and position yourself in the center of the screen to complete the recognition. Use your lower abdomen to lift your hips. Alternately, lift both feet and lower them to complete the movement. Exhale during the upward movement and inhale during the downward movement.
Module 5	Upper limb training module	Chest extension	Open your hands facing the screen to ensure your arms are fully visible. Straighten your arms to complete the stretch.
		Select up and down with straight arm	Stand sideways with your hands extended forward, then draw an arc with one hand from the front to the back, toward the top of your head.
		Grip push-ups in a kneeling position	Lie on your side facing the screen, with your hands supporting your body. Keep your body in a straight line from your shoulders to your knees. Inhale during the descent, with the chest reaching 2–3 cm above the ground, and exhale during the ascent.
		Push-up	Lie on your side facing the screen, with your hands supporting your body. Keep your body in a straight line from your shoulders to your ankles. Inhale during the descent, with the chest reaching 2–3 cm above the ground, and exhale during the ascent.
		Crouching rowing	Lean forward, avoid arching your back, and raise your hands from the floor to the back of your body, with your elbows extending beyond your body, feeling the contraction of your back.
		Cross decussation	Raise both hands and cross them over your forehead. When lowering, stretch both hands backward toward your body and feel the contraction of your back.

#### Embodied cognitive group (ECG)

The ECG program was delivered over 12 weeks as four 40-min face-to-face sessions per week in small school-based groups during scheduled activity periods. Sessions were delivered by trained facilitators following a standardized manual, with teachers assisting in supervision.

The intervention integrated socially interactive games with gross motor activities, framed within a narrative context (“Lizard Edi”) (Schmidt et al., 2020) to promote engagement in a low-threat, supportive environment. A three-stage progression was applied: Weeks 1–4 emphasized simple cue-based actions to reinforce attention and rule following, Weeks 5–8 introduced reversed or contradictory commands to increase cognitive control demands while normalizing errors, and Weeks 9–12 used subgroup-specific commands to increase exposure to peer observation and social evaluation in a structured manner. (Table 2; Figure 2).

**Table 2:** Progressive difficulty levels of six intervention games.

Game	Weeks 1–4 (low difficulty)	Weeks 5–8 (moderate difficulty)	Weeks 9–12 (high difficulty)
One Lizard, Two Lizards	Freeze at music stop using simple posture (e.g., sitting).	Stopping posture varies (e.g., one-legged stand, arms outstretched).	Must avoid repeating prior postures (e.g., crouching with both hands on the ground, extending one leg sideways)
Billy Billy Boo	Respond to “Billy Billy Boo” with one simple action (e.g., squat).	Inhibit response when signal is incomplete (“Billy Billy”).	Multiple cue–action pairs introduced and randomly alternated (e.g., “Billy Billy Boo” = squat, “Lizard Boo” = jump, “Billy Lizard” = spin once)
Lizard Song	Move in rhythm with music (e.g., crawling, kicking) and stop when paused.	Adapt to rhythm changes (slow vs. fast tempo).	Subgroups perform contrasting actions (e.g., crawling vs. kicking) in front of peers.
Wild Farm	Imitate animal movements or sounds; identify peers with the same role (e.g., hopping like a frog, neighing like a horse)	Combine both movements and sounds for increased complexity (e.g., hopping while making frog croaks, flapping arms while clucking like a chicken).	Roles switched unpredictably, requiring adaptation to rapid social changes (e.g., a child acting as a cow must suddenly switch to a duck)
Parking Timer	Run to music; stop at stimulus word and perform one designated action (e.g., squat).	Introduce multiple stimulus–response rules (e.g., “red light” = squat, “green light” = jump, “blue light” = clap)	Reverse stimulus–response mappings; errors normalized in playful context (e.g., “red light” now = jump, “green light” now = squat)
Disciplined Farmer	Act out animal roles following simple verbal commands (e.g., jumping, turning).	Visual cues (green/red cards) determine when to act (e.g., green card = act, red card = freeze).	Roles and visual signals are unpredictably reassigned, increasing exposure to social evaluation (e.g., a child playing “sheep” must suddenly switch to “dog” when a blue card appears).

Adherence to school-based sessions was documented using attendance records and instructor-completed session checklists to capture participation and protocol fidelity. A subsample of ECG participants also wore heart rate monitors and accelerometers during prespecified monitoring weeks for objective characterization of activity intensity and movement volume (Supplementary Table S1).

#### Combined intervention (CIG)

The CIG received a 12-week program delivered four times per week. Each session consisted of two sequential components: 20 min of classroom-based ECG delivered as described above, followed by 20 min of brief bouts of exercise delivered via the same mobile platform and structured according to the BBEG approach, with adaptations to accommodate the combined intervention format. Specifically, during each exercise session, all five training modules (stretching, lower-body, full-body, core, and upper-body training) were included; however, within each module, participants selected two exercises from the platform’s exercise library. This modification ensured comprehensive whole-body engagement while maintaining feasibility within the combined session duration.

The exercise component could be completed immediately following the school-based session or accumulated later on the same day at home or in the community. Adherence to the classroom-based component was documented using attendance records and instructor-completed checklists. Adherence to the home-based exercise component was tracked using platform-generated activity logs, supplemented by parent-assisted confirmation and post-session ratings of perceived exertion. Consistent with the BBEG condition, wearable heart rate monitors and accelerometers were applied only in a subsample of participants during prespecified monitoring weeks to provide periodic objective validation of exercise intensity and movement volume. Group-level physical activity validation indicators are reported in Supplementary Table S1 (see Figure 2).

#### Control group (CG)

Participants assigned to the Control Group did not receive any structured intervention during the 12-week study period. They were instructed to maintain their usual daily routines and physical activity levels. No cognitive training, exercise sessions, or additional behavioral programs were provided. Physical activity levels and extracurricular exposures were not systematically monitored in the control group, reflecting a usual-care condition in a real-world school setting.

### Measures

#### Questionnaire on demographic information

A self-designed questionnaire was employed to gather participants’ demographic information, encompassing factors such as gender, age, whether the child is a single child, parents’ employment outside the home, and the frequency of parents returning home.

#### Social anxiety symptoms

The validated Chinese version of the Social Anxiety Scale for Children (La Greca, 1999), previously validated in Chinese child populations (Fei), was administered at baseline, post-intervention, and at the 6-week follow-up. Social anxiety symptoms were assessed using the Social Anxiety Scale for Children (SASC) developed by La Greca (1999). The SASC is a 10-item self-report measure designed to evaluate social anxiety in children and adolescents. It comprises two subscales: Fear of Negative Evaluation (FNE) (e.g., “I am afraid of being teased”) and social avoidance and distress (SAD) (e.g., “I feel nervous when talking to unfamiliar children”). Each item is rated on a 3-point Likert scale ranging from 0 (never) to 2 (always), with higher scores indicating greater social anxiety. In the present study, the SASC demonstrated good internal consistency, with a Cronbach’s *α* coefficient of 0.892 for the total scale, and 0.823 and 0.716 for the FNE and SAD subscales, respectively. Based on the data collected in the current sample, the Kaiser Meyer Olkin (KMO) value was 0.729, as obtained from our own validation analysis, supporting the scale’s construct validity.

### Statistical analysis

All statistical analyzes were performed using IBM SPSS Statistics version 26.0 (IBM Corp., Armonk, NY, USA). Descriptive statistics were used to summarize participants’ baseline demographic and psychological characteristics. Continuous variables were reported as means and standard deviations (M ± SD), and categorical variables as frequencies and percentages. Baseline equivalence among groups was tested using independent-samples *t*-tests for continuous variables and chi-square (*χ*^2^) tests for categorical variables to ensure no significant differences prior to the intervention. To examine the effects of the intervention on social anxiety, a repeated-measures analysis of variance (ANOVA) was conducted, allowing for the evaluation of within-subject effects (changes over time), between-subject effects (differences among intervention groups), and the interaction effect between group and time. Assumptions of normality, sphericity, and homogeneity of variances were checked prior to analysis. When Mauchly’s test of sphericity was violated, Greenhouse–Geisser corrections were applied. The significance level was set at *p* < 0.05 (two-tailed) for all statistical tests.

## Results

### Baseline characteristics of participants

Table 3 summarizes the baseline characteristics of the four participant groups, including overall self-reported social anxiety and its subdimensions. Baseline comparisons indicated no significant differences among the four groups in total social anxiety scores or any of its dimensions (all *p* > 0.05), confirming group comparability prior to the intervention.

**Table 3:** Comparison of general characteristics among four groups [M ± SD, *n* (%)].

Variable	Characteristic	CG (*n* = 25)	BBEG (*n* = 25)	ECG (*n* = 25)	CIG (*n* = 25)	*F*/*χ^2^*	*p*
Gender	Male	16 (64%)	11 (44%)	11 (44%)	16 (64%)	1.34	0.26
	Female	9 (36%)	14 (56%)	14 (56%)	9 (36%)		
Age (year)		10.40 ± 0.82	10.20 ± 0.82	10.16 ± 0.80	10.68 ± 0.90	2.03	0.74
Single child	Single child	18 (72%)	17 (68%)	19 (76%)	18 (72%)	0.13	0.94
	Non-single child	7 (28%)	8 (32%)	6 (24%)	7 (28%)		
Parental outings	Father outing	14 (56%)	13 (52%)	13 (52%)	15 (60%)	0.60	0.91
	Mother’s outing	6 (24%)	6 (24%)	8 (32%)	4 (16%)		
	Parents’ outing	5 (20%)	6 (24%)	4 (16%)	6 (24%)		
Frequency of parental return home	1 time/week	2 (8%)	2 (8%)	2 (8%)	2 (8%)	0.33	0.92
	1 time/month	6 (24%)	7 (28%)	4 (16%)	4 (16%)		
	1 time/year	11 (44%)	11 (44%)	11 (44%)	15 (60%)		
	<1 time/year	6 (24%)	5 (20%)	8 (32%)	4 (16%)		
SA	Total score	10.60 ± 1.87	11.68 ± 3.29	10.40 ± 3.28	10.68 ± 3.01	0.96	0.42
	FNE	6.36 ± 1.60	7.00 ± 2.02	6.48 ± 1.91	5.84 ± 2.21	1.49	0.20
	SAD	4.76 ± 1.30	5.04 ± 1.93	5.24 ± 1.31	5.00 ± 1.58	0.18	0.91

CG, control group; BBEG, brief bouts of exercise group; ECG, embodied cognitive training group; CIG, combined intervention group; SA, social anxiety; FNE, fear of negative evaluation; SAD, social avoidance and distress. Baseline demographic characteristics refer to participants included in the final analytic sample (*n* = 100).

### Efficacy on social anxiety symptoms

Table 4 presents the means and standard deviations of total SA and its two subscales, FNE and SAD, across four groups at baseline (*T*_0_), after the intervention (*T*_1_), and at follow up (*T*_2_). A mixed design analysis of variance revealed significant Group by Time interactions for total SA, *F* (6, 232) = 10.49, *p* < 0.01, *ηp*^2^ = 0.25, indicating that the magnitude of change over time differed across groups. Significant interaction effects were also found for FNE, *F* (6, 232) = 4.06, *p* < 0.05, *ηp*^2^ = 0.11, and SAD, *F* (6, 232) = 4.16, *p* < 0.05, *ηp*^2^ = 0.14. Accordingly, follow-up simple effects analyzes were conducted. As shown in Supplementary Figure S1, scores generally declined over time in all intervention groups, although the trajectories differed by intervention.

**Table 4:** Effects of different interventions on social anxiety (M ± SD).

Variables	Group	*T*_0_	*T*_1_	*T*_2_	*F*/*η_p_*^2^/*p*_group_	*F*/*η_p_*^2^/*p*_time_	*F*/*η_p_*^2^/*p*_group*time_
SA	CG	10.60 ± 1.87	10.12 ± 2.53	10.34 ± 2.12	18.62/0.37/<0.01^**^	58.12/0.38/<0.01^**^	10.49/0.25/<0.01^**^
	BBEG	11.68 ± 3.29	7.10 ± 1.54^aa***^	5.50 ± 1.60 aa^***^			
	ECG	10.40 ± 3.28	8.30 ± 1.29^ab**^	7.60 ± 1.80^aab***^			
	CIG	10.68 ± 3.01	4.00 ± 1.35^aabbcc***^	2.80 ± 1.35^aabbcc***△△^			
FNE	CG	6.36 ± 1.60	7.24 ± 1.96	6.88 ± 1.69	11.39/0.26/<0.01^**^	41.78/0.30/<0.01^**^	4.06/0.11/<0.05^*^
	BBEG	7.00 ± 2.02	4.12 ± 0.97^aa**^	3.00 ± 1.73 aa^***^			
	ECG	6.48 ± 1.91	5.78 ± 1.83^ab*^	4.26 ± 1.08^aab***^			
	CIG	5.84 ± 2.21	2.40 ± 1.19^aabcc***^	1.18 ± 1.33^aabcc***△△^			
SAD	CG	4.76 ± 1.30	5.60 ± 0.96	5.20 ± 0.82	5.51/0.17/<0.05^*^	25.82/0.24<0.01^**^	4.16/0.14/<0.05^*^
	BBEG	5.04 ± 1.93	3.28 ± 1.35^aa**^	2.16 ± 1.09^aa***^			
	ECG	5.24 ± 1.31	4.24 ± 1.23^aab*^	3.56 ± 1.08^aab**^			
	CIG	5.00 ± 1.58	2.18 ± 0.91^aabcc***^	1.28 ± 1.11^aabcc***△△^			

a <0.05, aa <0.001, compared with CG; b <0.05, bb <0.001, compared with BBEG; c <0.05, cc <0.001, compared with ECG. ^*^p < 0.05. ^**^p < 0.01. ^***^p < 0.001, compared with *T*_0_; ^△△^*p* < 0.01, compared with *T*_1_.

Significant main effects of Time were observed for total SA, *F* (2, 232) = 58.12, *p* < 0.01, *ηp*^2^ = 0.38, as well as for FNE, *F* (2, 232) = 41.78, *p* < 0.01, *ηp*^2^ = 0.30, and SAD, *F* (2, 232) = 25.82, *p* < 0.01, *ηp*^2^ = 0.24. Simple effects analyses indicated that the CG showed no significant changes from *T*_0_ to *T*_1_ or *T*_2_ in total SA or either subscale (all *p* > 0.05), suggesting stable symptom levels across the assessment period. In contrast, all intervention groups showed significantly lower total SA, FNE, and SAD scores at both *T*_1_ and *T*_2_ than at *T*_0_ (all *p* < 0.05), indicating sustained improvements following the intervention. Further comparisons between *T*_1_ and *T*_2_ demonstrated additional significant reductions primarily in the CIG for total SA, FNE, and SAD (all *p* < 0.01), whereas changes from *T*_1_ to *T*_2_ were smaller and not consistently significant in the other intervention groups.

Significant main effects of Group were also observed for total SA, *F* (3, 116) = 18.62, *p* < 0.01, *ηp*^2^ = 0.37, for FNE, *F* (3, 116) = 11.39, *p* < 0.01, *ηp*^2^ = 0.26, and for SAD, *F* (3, 116) = 5.51, *p* < 0.05, *ηp*^2^ = 0.17. At baseline, no significant between group differences were detected for total SA, FNE, or SAD (all *p* > 0.05), supporting baseline comparability. At *T*_1_ and *T*_2_, the CG consistently showed higher total SA and subscale scores than all intervention groups (all *p* < 0.05). Moreover, the CIG demonstrated significantly lower total SA and FNE scores than both the BBEG and ECG at *T*_1_ and *T*_2_ (all *p* < 0.05), and it also showed lower SAD scores than the BBEG and ECG at *T*_1_ and *T*_2_ (*p* < 0.05). Comparisons between BBEG and ECG indicated that BBEG achieved lower scores than ECG at both *T*_1_ and *T*_2_ across total SA, FNE, and SAD (*p* < 0.05), although both groups improved relative to baseline.

## Discussion

Across outcomes of SA, FNE, and SAD, the present study demonstrated clear group-by-time differences following the interventions. All active intervention groups showed reductions in social anxiety–related outcomes relative to the control group, and these reductions were maintained at the 6-week follow-up. Importantly, the magnitude of change differed across interventions. For total SA, the CIG showed a larger absolute reduction from baseline to follow-up [Δ(*T*_0_–*T*_2_) = −7.88] than the BBEG [Δ(*T*_0_–*T*_2_) = −6.18] and the ECG [Δ(*T*_0_–*T*_2_) = −2.80] Similar patterns were observed for FNE and SAD, indicating consistent differences across core components of social anxiety. When considered in relation to prior research, these findings both align with and extend existing evidence. International studies and meta-analyzes typically report small to moderate reductions in anxiety symptoms following physical activity–based interventions in children and adolescents, particularly when interventions are brief and feasible to implement (Biddle and Asare, 2011; Rodriguez-Ayllon et al., 2019). The magnitude of change observed in BBEG in the present study falls within this commonly reported range, supporting the effectiveness of short bouts of physical activity for reducing anxiety-related outcomes (Lubans et al., 2016; Singh et al., 2023). Importantly, the larger reduction observed in the combined intervention suggests that integrating physical activity with structured cognitive–social practice may enhance intervention effects beyond those typically reported for single-modality approaches (Noda et al., 2024).

The embodied cognitive training intervention resulted in statistically significant, but comparatively smaller, reductions in social anxiety and its subdomains. In the present trial, the ECG group exhibited less absolute change than both the BBEG and CIG groups, indicating that cognitively oriented embodied practice may have limited impact when delivered in isolation. This pattern is consistent with theoretical accounts suggesting that embodied approaches can enhance attentional control, interoceptive awareness, and metacognitive monitoring, thereby attenuating automatic threat appraisal and social vigilance (Critchley and Garfinkel, 2018; Hofmann and Gómez, 2017; Noda et al., 2024; Tang et al., 2022). However, without concurrent physiological or behavioral activation, gains in cognitive regulation may be less likely to generalize to socially evaluative contexts characterized by heightened emotional arousal. In addition, embodied cognitive tasks alone may insufficiently target entrenched negative self-schemas or social evaluative biases unless paired with behavioral activation or exposure-based elements (Garland et al., 2011). This interpretation aligns with international evidence indicating that cognitively engaging and socially interactive interventions often yield modest effect sizes when broader behavioral or exposure components are absent (James et al., 2020; Weisz et al., 2017).

The brief bouts of exercise intervention showed larger short-term reductions in perceived social anxiety compared to embodied cognitive training, indicating that even brief and easily implemented bouts of physical activity can yield meaningful psychological benefits. This pattern is consistent with international evidence indicating that intermittent physical activity can enhance emotional well-being and reduce anxiety in children and adolescents (Lubans et al., 2016; Pascoe et al., 2020; Singh et al., 2025). In the present study, however, reductions observed in BBEG were smaller at follow-up than those observed in CIG, suggesting that behavioral activation alone may be insufficient to sustain improvements over time. Prior research indicates that longer-term maintenance of anxiety reduction often depends on addressing both behavioral and cognitive components, rather than relying on a single modality (Salmon, 2001).

Most importantly, the combined intervention integrating brief bouts of exercise with embodied cognitive training produced the largest absolute reductions across all outcomes. By directly comparing single-modality and combined interventions within the same randomized controlled trial, the present study provides evidence that integrating physical activity with cognitively oriented, socially embedded practice yields greater reductions in social anxiety than either approach alone. From a behavioral perspective, the combined intervention may exert its effects by concurrently engaging complementary regulatory processes. Brief physical activity primarily facilitates bottom-up modulation of arousal and stress responses, whereas embodied cognitive training targets top-down processes related to attention regulation, self-monitoring, and social interpretation. The simultaneous engagement of these processes may enhance self-regulatory capacity and promote more robust transfer of gains to everyday social contexts. This interpretation is consistent with prior research indicating that cognitively enriched movement interventions can improve executive functioning and emotional regulation more effectively than domain-specific training delivered in isolation (Mao et al., 2024). Crucially, the present study is innovative in demonstrating, under real-world and low-burden conditions, the synergistic effects of integrating brief bouts of exercise with embodied cognitive training on social anxiety in children. Supported by digital technology, the intervention enabled remote delivery, participant autonomy, and high adherence. Importantly, this integrated approach was applied to rural left-behind children, an underrepresented and high-risk population, highlighting its feasibility and scalability in resource-limited settings.

From a practical perspective, these findings have important implications. Because both intervention components are low-cost, time-efficient, and adaptable to existing school and home routines, the combined approach may be feasible for implementation in resource-limited rural settings. Embedding embodied cognitive activities within group-based school sessions, alongside encouraging brief physical activity outside the classroom, may offer a scalable and sustainable strategy for reducing social anxiety among left-behind children who have limited access to specialized psychological services.

### Limitations

Although the present study provides notable theoretical and practical implications, several limitations warrant recognition when interpreting and generalizing these findings. First, the sample was confined to left-behind children from one primary school in a single province, leading to geographic and socioeconomic homogeneity. This homogeneity constrains the generalizability of the findings to left-behind children across broader geographic regions and diverse socioeconomic backgrounds in China. Future studies should recruit more demographically and socioeconomically diverse samples to enhance external validity and facilitate the cross-context replicability of the observed effects.

Second, the assessment of physical activity participation in this study solely relied on on-site records by intervention staff and self-reports from participants. Although wearable heart rate monitors and accelerometers were used for a subset of participants during the scheduled monitoring period, continuous and full-course monitoring was not implemented for all participants, nor was continuous recording and quantitative analysis performed via continuous accelerometry, and future studies may adopt continuous accelerometry monitoring for the entire sample to strengthen the objectivity of the measurement results. In addition, all outcome measures were collected using self-report questionnaires completed by participants, which are inherently subject to subjectivity and response bias that cannot be fully eliminated even under researcher supervision, while physical activity during non-intervention periods was not systematically documented, making it impossible to effectively disentangle the independent effects of intervention-induced physical activity and spontaneous physical activity on the target outcomes. Future research may therefore implement multi-informant assessments involving multiple sources such as teachers and parents, combined with objective behavioral and physiological indicators for comprehensive measurement, to further improve the accuracy and comprehensiveness of outcome evaluation.

Third, no objective measures were implemented to monitor intervention adherence, hindering the accurate differentiation between superficial adherence and actual adherence among participants. Notably, adherence is not a dichotomous construct but rather exists on a continuum, encompassing partial adherence, occasional non-adherence, and complete non-adherence—a distinction that may result in the overestimation of short-term intervention effects and underestimation of long-term benefits. Furthermore, the unique characteristics of rural left-behind children—including their young age, insufficient family supervision, and limited access to professional physical activity guidance—may further compromise intervention implementation fidelity. Future research should therefore focus on identifying specific patterns of non-adherence and its key influencing factors, such as exercise motivation, family support, and academic stress.

Finally, participants in the control group were merely instructed to maintain their regular daily routines, with no systematic monitoring of their physical activity levels or extracurricular engagement. This lack of monitoring precluded the full exclusion of extraneous variance associated with unmeasured confounding activities, thereby undermining the study’s internal validity. Future studies may incorporate objective monitoring or activity logs for the control group to strengthen the reliability and robustness of study conclusions.

### Future directions

To address the aforementioned limitations of this study, existing evidence-based physical activity intervention studies have proposed several feasible and methodologically rigorous optimization strategies, which can serve as valuable references for future research. For physical activity monitoring, continuous wearable assessment using accelerometers (e.g., ActiGraph GT3X+) is recommended. This method allows for the objective quantification of participants’physical activity volume, intensity, and duration, and effectively reduces the subjective bias inherent in self-report measures (Lu et al., 2024; Smith et al., 2023). Complementary data collection via on-site observations by interventionists and weekly physical activity diaries can further improve the accuracy and integrity of monitoring data. With regard to verifying home-based physical activity implementation, and considering the unique characteristics of rural left-behind children, a multi-component verification framework of “remote parental supervision plus community volunteer follow-up” can be applied. This framework enables retrospective verification of home-based physical activity delivery through exercise video recordings and activity diary submissions. Simple tools such as mobile check-in mini-programs or paper-based checklists may also be used to enhance the traceability of physical activity behavior during non-intervention periods. Given the practical constraints in some rural regions, including low smartphone penetration and limited internet access, future studies should select context-appropriate monitoring and verification strategies. This approach will help maintain high data quality while ensuring the feasibility and practicability of the research design.

## Conclusion

This randomized controlled trial compared the effects of brief bouts of exercise, embodied cognitive training, and their combination on social anxiety among rural left-behind children. All three interventions were associated with reductions in social anxiety relative to the control group, with the combined intervention showing greater reductions than either single-modality intervention at post-intervention and at the 6-week follow-up. By directly comparing single and combined low-burden interventions within a real-world school and home context, this study extends existing evidence on accessible approaches to child social anxiety in an understudied population. These findings should be interpreted in light of the study design, including the single-site sample and relatively short follow-up period, and should therefore be viewed as preliminary comparative evidence rather than definitive conclusions regarding long-term effectiveness. From a practical perspective, a combined, low-cost intervention may represent a feasible option for reducing social anxiety in resource-limited settings, pending confirmation from future multi-site studies with longer follow-up.

## Data Availability

The raw data supporting the conclusions of this article will be made available by the authors, without undue reservation.
