# Correction: Effects of brief bouts of exercise, embodied cognitive training, and their combination on social anxiety in rural left-behind children: a randomized controlled trial

**DOI:** 10.3389/fpsyg.2026.1838841

**Published:** 2026-05-05

**Authors:** YiPing Luo, JiaXi Chen, Qian Yang, XiaoLin Li, Xiao Liu, WeiXin Dong, Jun Chen, ChunXia Lu

**Affiliations:** Department of Sport Education, Hunan Normal University, Changsha, Hunan, China

**Keywords:** brief bouts of exercise intervention, combined intervention, embodied cognitive training intervention, rural left-behind children, social anxiety

Author Jun Chen was erroneously omitted as corresponding author. The correct corresponding authors are Jun Chen and ChunXia Lu.

The original version of this article has been updated.

