# Supplementary figures and images for: Effects of brief bouts of exercise, embodied cognitive training, and their combination on social anxiety in rural left-behind children: a randomized controlled trial
Source: Front Psychol. 2026 Mar 17;17:1733845. doi: 10.3389/fpsyg.2026.1733845 (PMC13036845; doi:10.3389/fpsyg.2026.1733845)

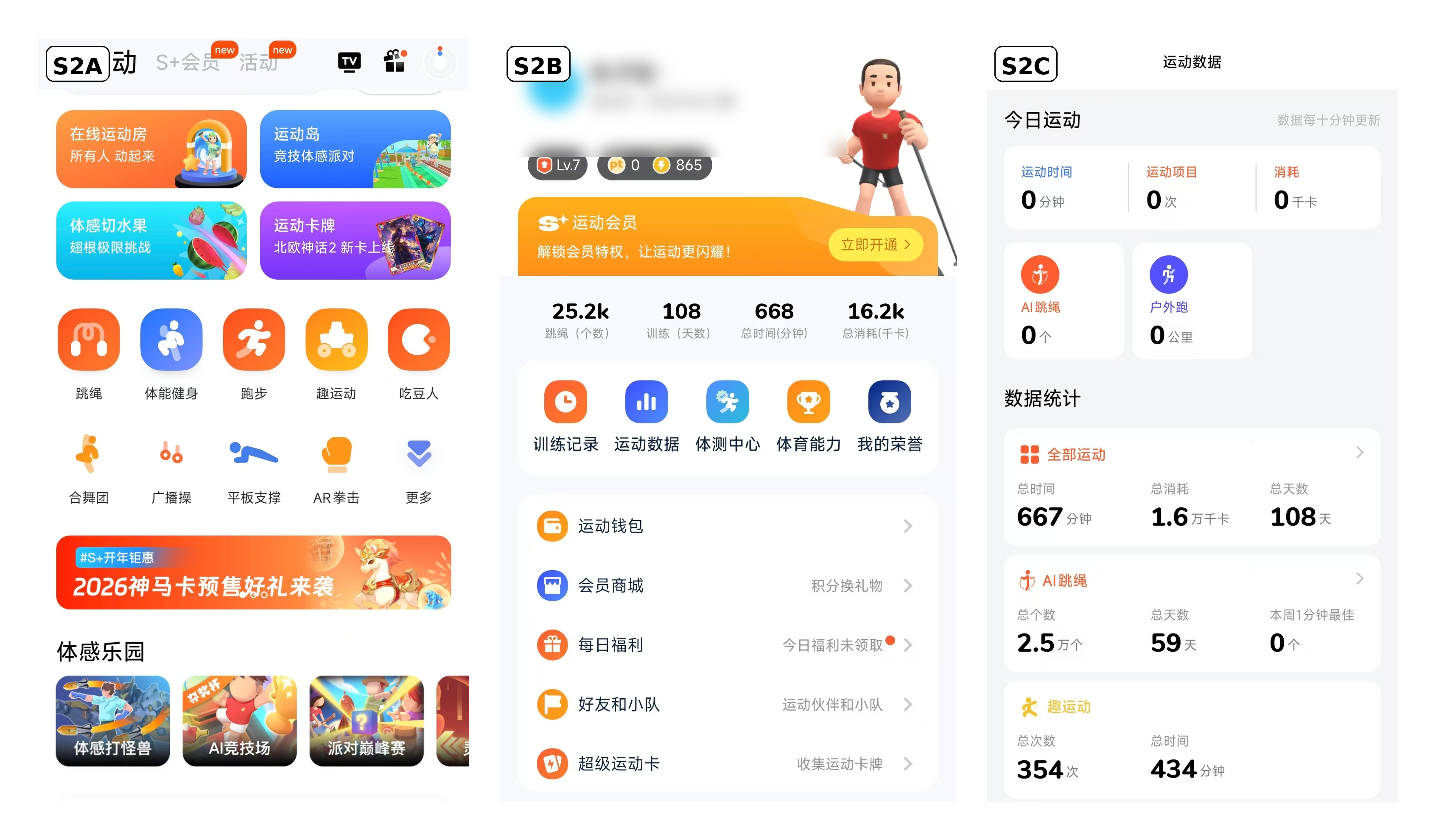

Supplement: Supplementary file 2 [file Image_2.png]
