# Supplementary material for: Effects of brief bouts of exercise, embodied cognitive training, and their combination on social anxiety in rural left-behind children: a randomized controlled trial
Source: Front Psychol. 2026 Mar 17;17:1733845. doi: 10.3389/fpsyg.2026.1733845 (PMC13036845; doi:10.3389/fpsyg.2026.1733845)
Supplement: Supplementary file 4 [file Table_1.docx]

**Supplementary Table S1**

| Indicator (Monitoring periods) | BBEG | ECG | CIG | CG |
| --- | --- | --- | --- | --- |
| Participants monitored, n | 25 | 25 | 25 | 25 |
| Monitoring occasions per participant, mean (SD) | 3.0 (0.0) | 3.0 (0.0) | 3.0 (0.0) | - |
| Total monitored sessions, n | 75 | 75 | 75 | - |
| Planned session duration (min) | 42.8 (3.5) | 39.2 (2.1) | 40.5 (2.8) | - |
| Mean HR (beats/min), mean (SD) | 146.3 (12.5) | 132.7 (10.8) | 141.5 (11.6) | - |
| Mean %HRmax, mean (SD) | 73.2 (4.8) | 66.4 (4.2) | 70.8 (4.5) | - |
| Peak %HRmax, mean (SD) | 78.9 (5.2) | 72.3 (4.6) | 76.5 (4.9) | - |
| Minutes in target zone, mean (SD) | 34.2 (5.8) | - | 31.5 (5.1) | - |
| Accelerometer wear time (min), mean (SD) | 128.5 (18.3) | 132.6 (16.7) | 130.2 (17.5) | - |
| MVPA minutes (min), mean (SD) | 40.3 (7.2) | 35.6 (6.5) | 38.2 (6.8) | - |
| RPE (Borg 6–20), mean (SD) | 14.8 (1.5) | 13.2 (1.3) | 14.1 (1.4) | - |

*Notes. Device-based validation was conducted only in a subsample of the active intervention groups (BBEG, ECG, and CIG) during prespecified monitoring weeks; therefore, no device indicators are reported for CG. Planned session duration refers to the scheduled intervention period, whereas accelerometer wear time reflects the total monitoring window and may exceed the session duration. Target zones were prespecified for BBEG and the exercise component of CIG only (65%–70% HRmax in Weeks 1–4, 70%–75% HRmax in Weeks 5–8, and 75%–80% HRmax in Weeks 9–12); thus, minutes in target zone are reported only for these groups.*
